# Supplementary material for: A comparative case study of the accommodation of students with disabilities in online and in-person degree programs
Source: PLoS One. 2023 Oct 12;18(10):e0288748. doi: 10.1371/journal.pone.0288748 (PMC10569535; doi:10.1371/journal.pone.0288748)
Supplement: S1 Table — (DOCX) [file pone.0288748.s001.docx]

**S1 Table. Student with disability’s demographics by modality**

|  | In-Person Students | Online Students |
| --- | --- | --- |
|  | N = 232^1^ | N = 126^1^ |
| Gender |  |  |
| Man | 59 (25.5%) | 22 (17.5%) |
| Woman | 172 (74.5%) | 104 (82.5%) |
| Decline to state | 1 | 0 |
| Race/Ethnicity |  |  |
| White or Asian | 164 (71.3%) | 74 (59.7%) |
| BLNP | 66 (28.7%) | 50 (40.3%) |
| Decline to state | 2 | 2 |
| Socioeconomic Status |  |  |
| Non-Pell Eligible | 121 (52.2%) | 47 (37.3%) |
| Pell Eligible | 111 (47.8%) | 79 (62.7%) |
| College Generation Status |  |  |
| Continuing Generation | 157 (67.7%) | 70 (55.6%) |
| First-Generation | 75 (32.3%) | 56 (44.4%) |
| Age in Years (continuous) | 20 (18–22) | 25.5 (22–29) |
| Decline to state | 2 | 0 |
| Age in Years (categorical) |  |  |
| Age ≤ 25 | 208 (90.4%) | 63 (50.0%) |
| Age > 25 | 22 (9.6%) | 63 (50.0%) |
| Decline to state | 2 | 0 |
| ^1^n (%); Median (IQR); Note: percentages shown exclude “decline to state” | | |
