# Supplementary material for: A comparative case study of the accommodation of students with disabilities in online and in-person degree programs
Source: PLoS One. 2023 Oct 12;18(10):e0288748. doi: 10.1371/journal.pone.0288748 (PMC10569535; doi:10.1371/journal.pone.0288748)
Supplement: S2 Table — (DOCX) [file pone.0288748.s002.docx]

**S2 Table. Difference in DRC enrollment by student demographics**

| Characteristic | OR^1^ | 95% CI^1^ | p-value |
| --- | --- | --- | --- |
| (Intercept) | 0.03 | 0.02, 0.03 | <0.001 |
| Campus |  |  |  |
| Online | — | — |  |
| In-Person | 1.70 | 1.46, 1.97 | <0.001 |
| Gender |  |  |  |
| Man | — | — |  |
| Woman | 2.37 | 2.05, 2.75 | <0.001 |
| College Generation Status |  |  |  |
| Continuing Generation | — | — |  |
| First-Generation | 0.78 | 0.69, 0.90 | <0.001 |
| Socioeconomic Status |  |  |  |
| Non-Pell Eligible | — | — |  |
| Pell Eligible | 1.20 | 1.05, 1.36 | 0.006 |
| Race/Ethnicity |  |  |  |
| White or Asian | — | — |  |
| BLNP | 1.03 | 0.90, 1.17 | 0.7 |
| Age in Years |  |  |  |
| Age ≤ 25 | — | — |  |
| Age > 25 | 1.44 | 1.22, 1.70 | <0.001 |
| Fewer than 30 Credit Hours | 0.89 | 0.74, 1.07 | 0.2 |
| ^1^OR = Odds Ratio, CI = Confidence Interval | | | |
