# Supplementary material for: A comparative case study of the accommodation of students with disabilities in online and in-person degree programs
Source: PLoS One. 2023 Oct 12;18(10):e0288748. doi: 10.1371/journal.pone.0288748 (PMC10569535; doi:10.1371/journal.pone.0288748)
Supplement: S3 Table — (DOCX) [file pone.0288748.s003.docx]

**S3 Table. Complete List of Accommodations in Dataset**

| Accessible Computer |
| --- |
| Alternative Testing [various]   - Alternative Testing Location - Computer - Memory Aid - Other - Reader - Scribe |
| Assistive Technology |
| Audio Recording |
| Chair |
| Conversion of written materials to be accessible by screen readers |
| Extra Time on Exams |
| Flexible Assignment Deadlines |
| Flexible Attendance |
| Lab Aide |
| Peer Notetaking Services |
| Powerpoints Notes and/or Other Lecture Materials in Advance |
| Preferential Seating |
| Realtime Captioning |
| Reduced Distraction Testing Environment |
| Service Animal |
| Sign Language Interpreting |
| Video Captioning (non-realtime) |
