# Supplementary material for: A comparative case study of the accommodation of students with disabilities in online and in-person degree programs
Source: PLoS One. 2023 Oct 12;18(10):e0288748. doi: 10.1371/journal.pone.0288748 (PMC10569535; doi:10.1371/journal.pone.0288748)
Supplement: S4 Table — (DOCX) [file pone.0288748.s004.docx]

**S4 Table. Regression results for disability type**

| Characteristic | Beta | 95% CI^1^ | p-value |
| --- | --- | --- | --- |
| (Intercept) | 0.63 | 0.50, 0.77 | <0.001 |
| GPAO | 0.69 | 0.66, 0.71 | <0.001 |
| Campus |  |  |  |
| Online | — | — |  |
| In-Person | 0.21 | 0.10, 0.33 | <0.001 |
| Gender |  |  |  |
| Man | — | — |  |
| Woman | -0.07 | -0.11, -0.03 | <0.001 |
| College Generation Status |  |  |  |
| Continuing Generation | — | — |  |
| First-Generation | -0.11 | -0.15, -0.07 | <0.001 |
| Socioeconomic Status |  |  |  |
| Non-Pell Eligible | — | — |  |
| Pell Eligible | -0.05 | -0.09, -0.01 | 0.010 |
| Race/Ethnicity |  |  |  |
| White or Asian | — | — |  |
| BLNP | -0.21 | -0.25, -0.17 | <0.001 |
| Age in Years |  |  |  |
| Age ≤ 25 | — | — |  |
| Age > 25 | 0.04 | -0.01, 0.09 | 0.12 |
| Fewer than 30 Credit Hours | -0.27 | -0.32, -0.21 | <0.001 |
| Disability Type |  |  |  |
| None | — | — |  |
| Learning disability | -0.06 | -0.29, 0.18 | 0.7 |
| Mental health/psychological disability | 0.07 | -0.15, 0.29 | 0.6 |
| Campus Interaction Effect |  |  |  |
| In-Person * Learning disability | -0.15 | -0.45, 0.15 | 0.3 |
| In-Person * Mental health/psychological disability | -0.22 | -0.48, 0.04 | 0.10 |
| ^1^CI = Confidence Interval | | | |
