# Supplementary material for: A comparative case study of the accommodation of students with disabilities in online and in-person degree programs
Source: PLoS One. 2023 Oct 12;18(10):e0288748. doi: 10.1371/journal.pone.0288748 (PMC10569535; doi:10.1371/journal.pone.0288748)
Supplement: Attachment — Submitted filename: Review Comments.docx [file pone.0288748.s005.docx]

1. Is the manuscript technically sound, and do the data support the conclusions?

The paper's abstract successfully meets the listed criteria. Firstly, it clearly outlines the study's primary objective, which is to compare disability accommodations in online and in-person biology degree programs. Secondly, it briefly explains how the study was conducted, including the selection criteria and statistical analyses utilized to address research questions. Thirdly, the abstract summarizes key findings, such as the superior academic performance of online students with disabilities compared to their in-person counterparts, despite the latter receiving more types of accommodations. It also emphasizes the need for institutions to modify accommodations to better support students in online courses. Finally, the abstract's length, at 284 words, falls within the prescribed 300-word limit.

The introduction of the document mostly meets the criteria listed, with a few exceptions. Firstly, it contextualizes the study and explains its significance by providing background information that notes the increasing popularity of online learning in higher education and the unique challenges faced by students with disabilities in such courses. Moreover, the problem addressed in the study, which is the potential mismatch between existing disability accommodations and the needs of students in online courses, is clearly defined. In addition, the introduction includes a brief review of the literature, citing previous studies that have examined disability accommodations in online courses. However, it does not mention any relevant controversies or disagreements in the field. The introduction concludes with a statement of the overall aim of the work, which is to compare the disability accommodations provided to students in online and in-person biology degree programs.

It appears that the authors have used a range of current and relevant references to support the theoretical justification of their study. The references cited include papers from academic journals, reports from national organizations, and books on disability studies and higher education. Many of the references are recent, with publication dates ranging from 2015 to 2023, indicating that the authors have made an effort to use up-to-date sources. Additionally, the references cover a range of topics related to disability and higher education, including academic performance, retention, accommodations, and online learning. Overall, it seems that the authors have used appropriate and relevant references to support the theoretical framework of their study.

The Materials and Methods section of the document provides sufficient detail to allow suitably skilled investigators to fully replicate the study. The authors describe the selection criteria for the courses analyzed, the data collection process, and the statistical analyses used to answer the research questions. They also provide a detailed description of the steps taken to protect the privacy and confidentiality of the students who participated in the study. The authors note that the data regarding student disability status and specific accommodation requests were stored separately from ordinary academic and demographic data, and that the identifiable disability data were handled only by staff members within the Disability Resource Center (DRC). The authors also provide a positionality statement that describes the backgrounds and experiences of the research team, which adds transparency to the research process.

The paper includes distinct Results, Discussion, and Conclusions sections that are well-structured and fulfill the criteria listed. In the Results section, the authors describe the findings of the study, including the differences in disability accommodations between online and face-to-face courses and the relationship between disability accommodations and academic performance. The authors present their results in tables and figures, which are clearly labelled and easy to understand. In the Discussion section, the authors the results of the study and places them in the context of previous research. The authors discuss the implications of their findings for institutions of higher education offering online courses and fully online degree programs, and suggest that institutions should examine and continuously monitor how their existing disability accommodations align with the needs of students in online courses and fully online degree programs. The authors also acknowledge the limitations of their study and suggest directions for future research. The Conclusions section summarizes the main findings of the study and reiterates the authors' recommendations for institutions of higher education. The authors note that their study provides important insights into the need for institutions to adapt their accommodations to better support students in online courses, and suggest that future research should focus on the experiences of students with disabilities in fully online degree programs.

Finally, the research complies with all applicable standards of experimental ethics and research integrity. The authors describe the measures they took to protect the privacy and confidentiality of the students who participated in the study. They also obtained institutional review board (IRB) approval from Arizona State University prior to conducting the research. The authors acknowledge the limitations of their study and caution against making generalisations about all people who share a specific type of disability or disability. Overall, the research appears to have been conducted in an ethical and responsible manner, with due attention to the privacy and well-being of the participants.

2. Has the **statistical analysis** been performed appropriately and rigorously?

Based on the information provided in the paper, the statistical analysis appears to be adequate. The authors used linear mixed effects regression to estimate the effect of student disability status on course grades, adjusting for the effects of prior academic performance, gender, race/ethnicity, age, college generation status, and socioeconomic status. They also used logistic regression to estimate the effects on Disability Resource Center (DRC) registration of degree program modality and possible interaction effects between modality and gender, race/ethnicity, college generation status, and socioeconomic status. The authors employed both forward selection and backward elimination for model selection. They also reported the beta coefficients, odds ratios, and 95% confidence intervals for each predictor variable. The authors acknowledged some limitations of the study, such as the use of administrative data and the possibility of bias due to the selection of the most well-informed students with disabilities.

3. Have the authors made all data underlying the findings in their manuscript fully available?

The paper does not explicitly state whether the data has been provided as part of the manuscript or its supporting information, or deposited to a public repository.

4. The paper is presented in an intelligible fashion and is written in standard English

The paper is presented in an intelligible fashion and is written in standard English. The authors use clear and concise language to describe their research methods, results, and conclusions. The paper is well-organized, with clear headings and subheadings that help readers navigate the content. The authors also provide definitions and explanations of technical terms and concepts, which makes the paper accessible to a broad audience. Overall, the paper is well-written and easy to understand, even for readers who may not be familiar with the specific field of study.

**What could be improved in the paper?**

Although it is difficult to identify specific improvements that could be made to the document as a whole, some general suggestions for improvement might include:

From the literature review standpoint, the paper could be improved in the following ways:

1. Expand the literature review: Although the paper provides a relevant literature review, it could be expanded to include more recent studies. It would be useful to include more recent studies that address the inclusion of students with disabilities in online programs.
2. Include studies that address the perspective of students with disabilities: The paper is based on administrative data and does not include the perspective of students with disabilities. It would be useful to include studies that address the experience of students with disabilities in online programs and how they feel supported and accommodated.

To make the research more clear, understandable, and replicable, the following improvements could be considered:

1. Describe the methodology used in more detail: Although the paper provides information on the methodology used, it could be described in more detail how the data was collected and analyzed. This would help readers better understand how the study's conclusions were reached.
2. Provide clear recommendations: The paper provides some recommendations for improving the inclusion of students with disabilities in postsecondary education, but more clear and specific recommendations could be provided for policymakers and education professionals.

It is important to note that, according to the Vancouver style for reference citation, it is necessary to review citations 15, 39, 42, 43, 46, 47, and 48. In these cases, it is possible that important information, such as the URL, volume and issue number, or DOI of the paper/paper, may be missing. Therefore, it is essential to carefully review each of these references to ensure that they conform to the required style."
