# Supplementary material for: A comparative case study of the accommodation of students with disabilities in online and in-person degree programs
Source: PLoS One. 2023 Oct 12;18(10):e0288748. doi: 10.1371/journal.pone.0288748 (PMC10569535; doi:10.1371/journal.pone.0288748)
Supplement: Attachment — Submitted filename: Response to reviewers.docx [file pone.0288748.s006.docx]

Journal Requirements:

When submitting your revision, we need you to address these additional requirements.

1. Please ensure that your manuscript meets PLOS ONE’s style requirements, including those for file naming. The PLOS ONE style templates can be found at https://journals.plos.org/plosone/s/file?id=wjVg/PLOSOne_formatting_sample_main_body.pdf and https://journals.plos.org/plosone/s/file?id=ba62/PLOSOne_formatting_sample_title_authors_affiliations.pdf

We have fixed the usage of “Fig.” to “Fig” in the main text and have changed the file naming for supplementary materials to match the guidance in those documents (i.e., each Supplemental Table is now in its own document titled S1 Table, S2 Table, etc.)

2. In your Data Availability statement, you have not specified where the minimal data set underlying the results described in your manuscript can be found. PLOS defines a study’s minimal data set as the underlying data used to reach the conclusions drawn in the manuscript and any additional data required to replicate the reported study findings in their entirety. All PLOS journals require that the minimal data set be made fully available. For more information about our data policy, please see http://journals.plos.org/plosone/s/data-availability.

“Upon re-submitting your revised manuscript, please upload your study’s minimal underlying data set as either Supporting Information files or to a stable, public repository and include the relevant URLs, DOIs, or accession numbers within your revised cover letter. For a list of acceptable repositories, please see http://journals.plos.org/plosone/s/data-availability#loc-recommended-repositories. Any potentially identifying patient information must be fully anonymized.

Important: If there are ethical or legal restrictions to sharing your data publicly, please explain these restrictions in detail. Please see our guidelines for more information on what we consider unacceptable restrictions to publicly sharing data: http://journals.plos.org/plosone/s/data-availability#loc-unacceptable-data-access-restrictions. Note that it is not acceptable for the authors to be the sole named individuals responsible for ensuring data access.

We will update your Data Availability statement to reflect the information you provide in your cover letter.

We have changed the data availability statement in the online submission tool. We also describe our ethical concerns regarding making the raw data available in our cover letter. The data availability statement is also reproduced here: “The data analyzed in this study describe students’ individual disability statuses. They also include enough demographic information to make re-identification of individuals possible. For these reasons, it is not possible to publicly release the raw data. Qualified researchers may request access through https://uoia.asu.edu/contact.”

3. Please include your full ethics statement in the ‘Methods’ section of your manuscript file. In your statement, please include the full name of the IRB or ethics committee who approved or waived your study, as well as whether or not you obtained informed written or verbal consent. If consent was waived for your study, please include this information in your statement as well.

This statement has been updated as requested.

4. Please review your reference list to ensure that it is complete and correct. If you have cited papers that have been retracted, please include the rationale for doing so in the manuscript text, or remove these references and replace them with relevant current references. Any changes to the reference list should be mentioned in the rebuttal letter that accompanies your revised manuscript. If you need to cite a retracted article, indicate the article’s retracted status in the References list and also include a citation and full reference for the retraction notice.

As described below, we reviewed the references list, particularly those listed by Reviewer 1 as missing required elements. Please note that these changes cannot be tracked using MS Word’s tracked changes feature.

Additional Editor Comments: The reviewers agreed on the high quality of the submitted manuscript and cited its importance. The reviewers pointed out possible corrections that would improve the text, which I, as editor, also agree with. Therefore, I would like to ask you to address the reviewers’ comments or argue why making changes is not possible.

[Note: HTML markup is below. Please do not edit.]

Reviewers’ comments:

# Reviewer 1

I have had the privilege of reviewing your paper titled “Comparing Disability Accommodations in Online and In-Person Biology Degree Programs”. As a reviewer, my goal is to help improve the quality of the paper so that it is suitable for publication in the PLOS ONE Journal. To this end, I have provided a detailed evaluation of your work in the attached “Reviewer’s Comments” file. I hope these observations will be useful to you in reviewing and improving your manuscript. Please note that my comments are offered with the intention of being constructive and improving the quality of the work.

[We have excerpted specific requests from Reviewer 1’s attached document]

However, it does not mention any relevant controversies or disagreements in the field.

Respectfully, we believe that the introduction highlights open questions from the previous literature as they relate to the focus of our study. These include the many issues that were raised during the COVID-19 pandemic and the resultant shift to remote learning. They also include the fundamental uncertainty, owing to the novelty of these programs, regarding how students with disabilities are supported in online programs. We do also note uncertainty or research disagreements about the value of specific types of accommodation, e.g., separate room testing.

From the literature review standpoint, the paper could be improved in the following ways:

1. Expand the literature review: Although the paper provides a relevant literature review, it could be expanded to include more recent studies. It would be useful to include more recent studies that address the inclusion of students with disabilities in online programs.

As we describe in the introduction, owing to the novelty of fully online degree programs, there are no previous studies of this specific issue. We do, however, cite and review the recent literature on the experiences of students with disabilities in online courses.

2. Include studies that address the perspective of students with disabilities: The paper is based on administrative data and does not include the perspective of students with disabilities. It would be useful to include studies that address the experience of students with disabilities in online programs and how they feel supported and accommodated.

While we agree that this is an important topic (and indeed we note this as a limitation), addressing this would require a new and separate research study.

To make the research more clear, understandable, and replicable, the following improvements could be considered:

1. Describe the methodology used in more detail: Although the paper provides information on the methodology used, it could be described in more detail how the data was collected and analyzed. This would help readers better understand how the study’s conclusions were reached.

We reviewed this section and we believe that the existing description is both clear and complete. Since the reviewer did not provide specific examples of which aspects of this section they felt were lacking in detail, we would prefer to leave the text as is.

2. Provide clear recommendations: The paper provides some recommendations for improving the inclusion of students with disabilities in postsecondary education, but more clear and specific recommendations could be provided for policymakers and education professionals.

Thank you. We have revised the final section to make our recommendations clearer.

It is important to note that, according to the Vancouver style for reference citation, it is necessary to review citations 15, 39, 42, 43, 46, 47, and 48. In these cases, it is possible that important information, such as the URL, volume and issue number, or DOI of the paper/paper, may be missing. Therefore, it is essential to carefully review each of these references to ensure that they conform to the required style.”

We have examined the listed references. 15, 39, 42, 47, 48 are missing certain information but these fields are not available from the journals (generally doi). 43, 46 were missing available information and we have added it.

# Reviewer 2

1. Please explain the inconsistence in the information in Ethics Statement (Consent was not obtained because the data were analyzed anonymously. The work was done under a protocol approved by the ASU IRB. This is stated in the manuscript) and Data Availability Section (The data analyzed in this study involve students’ individual disability statuses and are considered very sensitive. We were granted permission to analyze them, but do not have permission to disseminate the data themselves). This is explained in details in the paper (line 182 and next), but need to be clarify in the Additional information section.

See comments above and in the cover letter.

2. There is a huge discrepancy in number of students using notetaking services (Table 5. Percentage of accommodation-eligible students receiving common accommodations by mode - line 344). Did you check if the reason is using notetaking (dictating) software instead notetaking services by online students?

Thank you for this question. The “notetaking service” in that table and in the text would be more clearly labeled “peer notetaking services”. So, this is in fact a fellow classmate who agrees to (and is compensated for) sharing notes or some equivalent arrangement with the student receiving the accommodation. This is, therefore, related to but distinct from a dictation software, which is a separate accommodation. We have changed the name of this accommodation throughout the text and added some explanatory text to emphasize the nature of this accommodation.

3. I really appreciate the analysis of factors related to how students with disabilities approach programs (line 412 and next), however in the part Willingness to request (line 471 and next) please consider if one of the reason is lover pressure of the environment during online classes. Visibility of students difficulties and needs during online classes is much more difficult to recognize by both teachers and peers and therefore fostering to disclosure is significantly lower in this group.

Thank you. We have added a sentence to the “willingness to request” section that we hope captures this idea.

4. Another possible factor to analyze is higher motivation of some students with disabilities to study online because it seems to be easier option to them. Are students with ASD more willing to online studies because of their communication difficulties? Are students with motor difficulties or blindness more willing to online studies because of their mobility difficulties?

This is similar to something that we do address (in the “Need” paragraph), but your comment suggests that we should be clearer in our language there. We have edited that paragraph to explain what mechanism we propose maybe at work.

5. Please check „orphans” in text editing, i.e. line 318-319, 381, 389.

Thank you. It is our understanding that these will be addressed in final typesetting, but we will keep an eye out.

While revising your submission, please upload your figure files to the Preflight Analysis and Conversion Engine (PACE) digital diagnostic tool, https://pacev2.apexcovantage.com/. PACE helps ensure that figures meet PLOS requirements. To use PACE, you must first register as a user. Registration is free. Then, login and navigate to the UPLOAD tab, where you will find detailed instructions on how to use the tool. If you encounter any issues or have any questions when using PACE, please email PLOS at figures@plos.org. Please note that Supporting Information files do not need this step.

We have processed our Figure through PACE as requested.
